# International outbreak of *Salmonella *Oranienburg due to German chocolate

**DOI:** 10.1186/1471-2334-5-7

**Published:** 2005-02-03

**Authors:** Dirk Werber, Johannes Dreesman, Fabian Feil, Ulrich van Treeck, Gerhard Fell, Steen Ethelberg, Anja M Hauri, Peter Roggentin, Rita Prager, Ian ST Fisher, Susanne C Behnke, Edda Bartelt, Ekkehard Weise, Andrea Ellis, Anja Siitonen, Yvonne Andersson, Helmut Tschäpe, Michael H Kramer, Andrea Ammon

**Affiliations:** 1 Department of Infectious Disease Epidemiology, Robert Koch-Institut, Berlin, Germany; 2 Niedersächsisches Landesgesundheitsamt, Hannover, Germany; 3 Landesinstitut für den öffentlichen Gesundheitsdienst, Nordrhein-Westfalen, Germany; 4 Institute for Hygiene and Environment, Center for Infectious Disease Epidemiology, Germany; 5 Department of Bacteriology, Mycology and Parasitology, Statens Serum Institut, Copenhagen, Denmark; 6 Government Health Service Institute, Dillenburg, Germany; 7 National Reference Centre for Salmonella and other Bacterial Enteric Pathogens, Institute for Hygiene and Environment Hamburg, Germany; 8 National Reference Centre for Salmonella and other Bacterial Enteric Pathogens, Robert Koch-Institut, Wernigerode, Germany; 9 Enter-net surveillance hub, HPA Communicable Disease Surveillance Centre, London, United Kingdom; 10 Federal Institute for Risk Assessment, Berlin, Germany; 11 Foodborne, Waterborne and Zoonotic Diseases Division, PPHB, Health Canada; 12 Laboratory of Enteric Pathogens, Department of Microbiology, Helsinki, Finland; 13 Swedish Institute for Infectious Disease Control, Stockholm, Sweden

## Abstract

**Background:**

This report describes a large international chocolate-associated Salmonella outbreak originating from Germany.

**Methods:**

We conducted epidemiologic investigations including a case-control study, and food safety investigations. *Salmonella *(*S*.) Oranienburg isolates were subtyped by the use of pulsed-field gel electrophoresis (PFGE).

**Results:**

From 1 October 2001 through 24 March 2002, an estimated excess of 439 *S*. Oranienburg notifications was registered in Germany. Simultaneously, an increase in *S*. Oranienburg infections was noted in other European countries in the Enter-net surveillance network. In a multistate matched case-control study in Germany, daily consumption of chocolate (matched odds ratio [MOR]: 4.8; 95% confidence interval [CI]: 1.3–26.5), having shopped at a large chain of discount grocery stores (MOR: 4.2; CI: 1.2–23.0), and consumption of chocolate purchased there (MOR: 5.0; CI: 1.1–47.0) were associated with illness. Subsequently, two brands from the same company, one exclusively produced for that chain, tested positive for *S*. Oranienburg. In two other European countries and in Canada chocolate from company A was ascertained that also contained *S*. Oranienburg. Isolates from humans and from chocolates had indistinguishable PFGE profiles. No source or point of contamination was identified. Epidemiological identification of chocolate as a vehicle of infections required two months, and was facilitated by proxy measures.

**Conclusions:**

Despite the use of improved production technologies, the chocolate industry continues to carry a small risk of manufacturing *Salmonella*-containing products. Particularly in diffuse outbreak-settings, clear associations with surrogates of exposure should suffice to trigger public health action. Networks such as Enter-net have become invaluable for facilitating rapid and appropriate management of international outbreaks.

## Background

Non-typhoidal *Salmonella *spp. is a substantive cause of human gastroenteritis in many parts of the world [1]. In Germany, non-typhoidal salmonellosis remains the most frequently reported infectious disease. For example, in 2001, the 77,185 *Salmonella *reports (incidence:94/100,000) received at the federal level by the Robert Koch-Institut (RKI) accounted for 31% of all notifications for the 54 notifiable conditions [2]. *Salmonella enterica *subspecies *enterica *serotype Enteritidis (*S*. Enteritidis) is the predominating serotype followed by *S*. Typhimurium. They represented 65% and 23% of the reported cases of non-typhoidal salmonelloses with known serotype in 2001. Thus, the remaining ~250 serotypes reported to the RKI in that year, including S. Oranienburg, accounted for only 12%.

In mid-October 2001, the National Reference Center for *Salmonella *and Other Enteric Pathogens (NRC) in Hamburg noted an unusual increase in the number of *S*. Oranienburg isolates received in October. At that time, no increase was noticeable in the national database for statutorily reportable infectious diseases; 50 *S*. Oranienburg notifications (median: 1 per week) had been registered for 2001. On November 19, the NRC in Wernigerode informed the RKI that it had received a *S*. Oranienburg isolate in September. The isolate was submitted by a private laboratory for serotyping and had come with the additional source information "confectionery sample". Upon inquiry, a large German chocolate manufacturer (company A), which produced a broad variety of chocolates and products made thereof, called the RKI on November 27, and confirmed that it had sent in the confectionery sample. According to company A, the positive sample originated from an in-house control of a chocolate product and the pertaining batch, due to be exported to the United States, was completely destroyed and not distributed. Notwithstanding, the number of statutory *S*. Oranienburg notifications had sharply increased and continued to rise. This report describes the epidemiologic, food safety, and microbiological investigations of this outbreak.

## Methods

### Epidemiologic investigation

#### Descriptive epidemiology

A standard exploratory questionnaire was distributed on 20 November 2001 via state health departments to all local health departments to aid the collection of data on food and environmental exposure from cases. In addition, local health departments were asked to immediately interview patients with newly reported *S*. Oranienburg infections about chocolate consumption in the seven days before disease onset, and also to send any remaining chocolate to a food safety laboratory.

#### International case-finding

A request was distributed to participants of the Enter-net surveillance network [6] on December 10, to see if other countries were affected or had relevant information.

#### Case-control study

On December 3, while exploration of patients were ongoing and results inconclusive, *S*. Oranienburg isolates from patients and from the in-house chocolate control were found to be indistinguishable by pulsed-field gel electrophoresis (PFGE). On the same day, a multistate case-control study was initiated and coordinated by RKI to test the hypothesis that at least one product from company A was associated with *S*. Oranienburg-infections. As we were denied a product list from company A, we resorted to the company's web-site and included in our food history evaluation all the products listed there. Some products from company A, e.g., bars of chocolate (brand A), were exclusively sold at a large chain of discount grocery stores (chain X). We found that the majority of chocolates sold at chain X were produced by company A. Therefore, for the analysis we constructed a variable for chocolate(s) purchased at chain X ("chain-X-chocolate") as a proxy for chocolate-products from company A because most patients could remember the flavor of the purchased chocolate, but seldom the brand name.

The hypothesis-testing questionnaire collected data on the consumption of chocolates, and some other foods, particularly those previously associated with outbreaks of *S*. Oranienburg in other countries [3-5]. Food-consumption history was evaluated for two different time periods, i.e., for the seven days prior to onset of symptoms in the case-patients and for the seven days before the interview. In addition, case-patients were asked about clinical symptoms, duration of illness and hospitalization. We defined a case-patient as a person with gastroenteritis starting after 1 October 2001 who had been reported with a *S*. Oranienburg infection to a public health department before December 6. Case-patients were excluded from the analysis, if they could have been secondary, i.e., if they reported to having had contact with a person with diarrhea in the seven days prior to symptom onset. Cases were selected from the national reportable database by simple random sampling; in Lower Saxony an attempt to interview all case-patients was launched. Case selection was done irrespective of whether patients also had been interviewed with an exploratory questionnaire. At least one age and telephone exchange-matched control subject was selected for each case-patient by sequentially adding 2 to each case-patients' telephone number. Control subjects were eligible if they were in the same age-group as their matched case-patient (0–5 years, 6–17 years, 18–59 years, 60 years or older), had no gastrointestinal symptoms after 1 October 2001, and had not traveled abroad in the seven days prior to the onset of symptoms of the matched case. Telephone interviews were conducted by state health departments, local health departments, and the RKI. Data were analyzed with Epi Info V6.04c (Centers for Disease Control and Prevention, Atlanta, GA).

### Investigation by the food safety authorities

The local food safety authority inspected company A's production facility and took samples from already packaged ("in-house") chocolates, and from ingredients from its suppliers. Beginning December 11, a nationwide chocolate sampling of German chocolates in grocery stores was initiated by the Federal Ministry of Consumer Protection, Food and Agriculture, and was assisted by the Federal Institute for Risk Assessment (Bundesinstitut für Risikobewertung, "BfR"). On December 18, when a chocolate leftover from brand A tested positive, the investigations were tailored to German chocolates from company A. The BfR examined quantitatively four *Salmonella *positive chocolate leftovers and five chocolates from grocery stores using the most probable number technique [7].

### Molecular subtyping

For comparison by the use of PFGE, *S*. Oranienburg isolated from stool specimens were sent to the NRC from laboratories in Germany and, on Enter-net request, from other countries. Furthermore, isolates from chocolates were submitted from state or private food laboratories in Germany as well as from Canada and the Czech Republic. PFGE-analysis was carried out according to Prager et al [8].

## Results

### Epidemiologic investigation

#### Descriptive epidemiology

**Figure 1:**
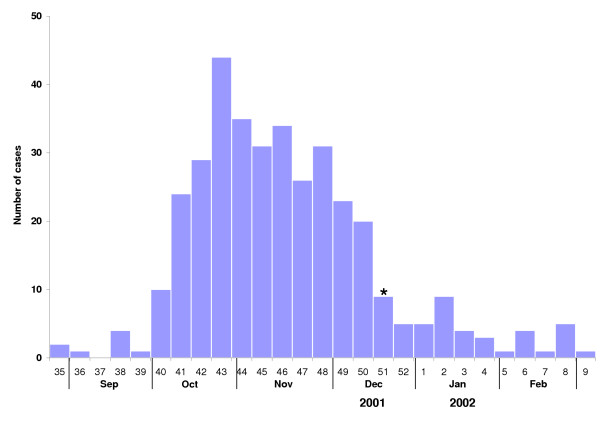
Disease onset (n = 362) of reported (n = 462) *S*. Oranienburg cases from reporting week 42/2001 to reporting week 12/2002 (outbreak period). The asterisk indicates the week when the (first) public warning was issued, and the incriminated chocolate products were recalled

In 2001, the RKI received 50 reports of *S*. Oranienburg up to reporting week 42, but 462 reports in the following 23 weeks (15 October 2001–24 March 2002, "outbreak period", figure 1). Thus, an excess of 439 *S*. Oranienburg reports were registered assuming a background rate of one report per week. The median age was 15 years (range: 0–92 yrs), 240 (52%) patients were female. There was no difference in the gender distribution within the single 10-year age-bands (*P *= .51). All 16 states of Germany reported *S*. Oranienburg cases during the outbreak period, with the highest incidence in the state of Schleswig-Holstein (1.78/100,000) bordering on Denmark. In total, 206 of the 440 German counties were affected with a median of one report and a maximum of 16 from the city of Hamburg during the outbreak period.

Sixty exploratory questionnaires were received from eight states by the end of 2001. Forty-three (88%) of 49 patients with information on chocolate consumption had a symptom onset after 1 October 2001. Of the 34 who gave information as to where they bought the chocolate, 21 (62%) explicitly reported chain X. Some reported exclusively having eaten chocolate bars from brand A, among them a two-year-old child. On 18 December 2001, two months after the initial outbreak alert, a leftover consumed reportedly by this child in the seven days before symptom onset tested positive for *S*. Oranienburg.

#### International case-finding

**Figure 2:**
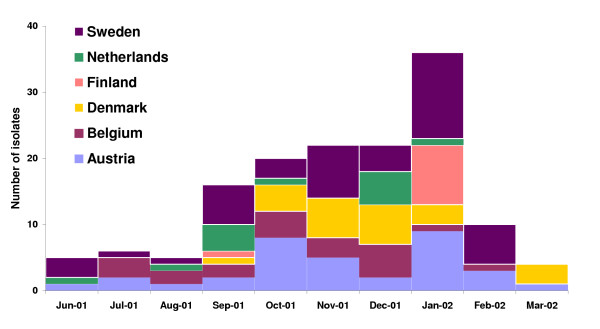
Number of *S*. Oranienburg infections reported to the Enter-net database from participating countries, except Germany

On December 11, one day after the Enter-net request was distributed, Denmark was the first country to respond. Twelve cases of *S*. Oranienburg had been reported in Denmark from October 18 through December 10, compared with only two cases in 2001 before October 18. None of the clustered cases were travel-related [9]. Exploratory patient interviews had already been conducted at the time of the Enter-net request. At this point in time the investigators in Denmark, without knowledge of the German *S*. Oranienburg problem, independently suspected German chocolate bought in chain X as the source of the Danish outbreak. Chocolate was the only food item that all patients reported eating. The majority stated purchasing chocolate in chain X, which, although German, operates internationally [9]. In the next few days, an increase in the number of *S*. Oranienburg infections was reported from other countries such as Austria, Belgium, Finland, Sweden, The Netherlands (figure 2), and Canada [10]. As it became apparent that German chocolate was contaminated with *S*. Oranienburg, patient interviews were conducted that showed that several patients remembered having consumed German chocolate [10], except in Canada where all of the patients denied this consumption.

#### Case-control study

Sixty cases and 62 controls from five states were enrolled in the matched case-control study. Interviews were conducted with a median delay of 37 days (range: 12–64 days) after disease onset in case-patients. Twelve case-control pairs were excluded from chocolate-specific analysis, nine because the case-patients could have been secondary, one due to illness in September, and two where the control subjects could not remember whether they had eaten chocolate. Of the 48 cases and 50 controls that were analyzed, 24 (50%) case-patients and 32 (67%) control subjects were female, 22 (46%) case-patients were younger than 10 years. Ten (21%) of the case-patients reported to have suffered from bloody diarrhea and 14 (29%) were hospitalized (table 1). Results of the preliminary analysis were available on December 14. All 48 case-patients ate chocolate in the seven days before symptom onset, but this also applied to 43 (86%) of the control subjects. Three variables relating to the seven-day period prior to symptom onset of the case-patient were significantly associated with disease (table 2). The first variable was having shopped at chain X (matched odds ratio [MOR]: 4.2; 95% confidence interval [CI]: 1.2–23.0). The second variable was having consumed chain-X-chocolate (MOR: 5.0; 95% CI: 1.1–47.0). Eleven (25%) of 44 case-patients gave such an exposure history, six of whom reported having consumed either brand A chocolate exclusively (which could be inferred from the flavor of chocolate eaten), or were uncertain whether they had also eaten another kind of chocolate (n = 3). The third variable was having eaten (any kind of) chocolate on a daily basis (MOR: 4.8; 95% CI: 1.3–26.5). None of the other variables including all those relating to the seven days before the interview were significantly associated with illness.

**Table 1:** Clinical characteristics of *S*. Oranienburg cases (n = 48) analyzed in a case-control-study, December 2001

Symptoms	Frequency, n (%)
Diarrhea	41 (85)
Fever > 38,5°C	27 (56)
Vomiting	17 (35)
Hospitalization	14 (29)
Antimicrobial medication	12 (25)
Visible blood in stool	10 (21)

**Table 2:** Significant risk factors for *S*. Oranienburg-associated illness in Germany, October-December 2001

Exposure	Cases exposed (n/N, %) *	Controls exposed (n/N, %)*	MOR	Exact 95% CI	*P*-value
Ate chocolate bought at chain X	11/44 (25)	2/45 (4)	5.0	1.1, 47.0	0.04
Daily consumption of chocolate	22/48 (46)	12/50 (24)	4.8	1.3, 26.5	0.01
Shopped at chain X	31/44 (71)	19/45 (42)	4.2	1.2, 23.0	0.03

MOR = Matched odds ratio, CI = Confidence interval

* Proportion and percentages of cases and controls exposed ignoring matching

#### Public health action

On December 18, the finding of *S*. Oranienburg in a chocolate leftover of a patient led to an immediate public warning and recall of all chocolates of this brand with specific production numbers by company A. The recall was extended to other products from company A a few days later. Chocolates included in the German recall were promptly withdrawn from the market in other European countries as well as in Canada. In Canada, Finland, and Sweden, samples from withdrawn chocolates tested positive for *S*. Oranienburg [10].

### Investigation by the food safety authorities

The local food safety authority in Germany did not identify hygienic deficiencies at the production facility. Samples obtained in the beginning of December 2001 from in-house chocolates (n = 12), as well as from cocoa (n = 3) and cocoa powder (n = 7) from a supplier of company A tested negative. This applied also to German chocolates sampled in grocery stores until 18 December (on that day a leftover tested positive). Overall, *S*. Oranienburg was found in 18 (5%) of 381 chocolates that were tested and reported to BfR during the outbreak period. *S*. Oranienburg was isolated from two different brands of company A; all positive chocolates were produced during the same week in August 2001. Estimates of the number of *Salmonella *in the tested chocolates ranged between 1.1 and 2.8 per gram.

### Molecular subtyping

**Figure 3:**
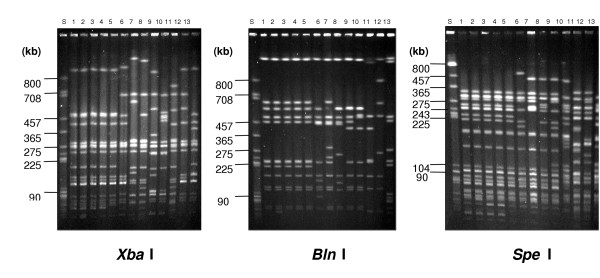
Comparison of human *S*. Oranienburg isolates from the outbreak- period with strains of this serovar received sporadically before the outbreak by the use of PFGE (digested with *Xba*I, *Bln*I, and *Spe*I) lanes: 1–5: isolates from the outbreak period 6–13: isolates before the outbreak period S: molecular reference

From October 2001 through January 2002, the NRC received 98 *S*. Oranienburg isolates from human cases of gastroenteritis originating in Germany (n = 52), Austria (n = 19), Belgium (n = 8), Canada (n = 6), Denmark (n = 4), The Netherlands (n = 4), Sweden (n = 4), and the Czech Republic (n = 1). Furthermore, 15 chocolate isolates were sent to the NRC for PFGE-analysis from Germany (n = 12), Canada (n = 2), and the Czech Republic (n = 1). They came from an in-house sample, from leftovers of chocolates consumed by patients in their incubation period, and from chocolates sampled in grocery stores in Germany. The PFGE profiles of *S*. Oranienburg isolates from patients with symptom onset after 1 October 2001 (outbreak period) in Germany and in the other countries mentioned above, except Canada, were indistinguishable (figure 3), but differed from *S*. Oranienburg isolates from German patients with symptom onset before October. All 15 chocolate isolates showed PFGE profiles indistinguishable from human isolates of the outbreak period.

## Discussion

We describe an international outbreak of *S*. Oranienburg and present several lines of evidence that German chocolate from company A was the vehicle of infections. *S*. Oranienburg, a rare serotype in food as well as in humans in Germany, was isolated from retail-sampled chocolates of two brands produced by company A, from chocolate leftovers that had been consumed by patients before symptom onset, and from an in-house sample of company A obtained prior to the outbreak. In a case-control study, *S*. Oranienburg infection was significantly associated with the consumption of chain-X-chocolate (proxy for chocolate from company A) in the week prior to symptom onset, but not in the seven days before the interview. Case-patients were more likely than control subjects to report eating chocolate daily, likely indicating an increased probability of having been exposed to contaminated chocolate. Furthermore, patient isolates from the outbreak period shared a PFGE profile with isolates from chocolates but differed from isolates of patients who became sporadically diseased with *S*. Oranienburg before the outbreak. In addition, the food histories and microbiological results from *S*. Oranienburg patients in several other countries pointed to the same source [9,10].

*Salmonella *infections after consumption of contaminated chocolate, although rare, have been known since the 1960's [11]. Common to all reported chocolate-outbreaks, including ours, was that the epidemics were propagated in time, widely disseminated geographically, and affected large number of persons, predominantly children [12-17] (table 3). In addition, only very small numbers of *Salmonella *have been recovered from chocolates in these outbreaks, suggesting a very low infectious dose. Estimates of the number of *S*. Oranienburg cells per gram in this outbreak ranged from 1.1–2.8. However, we cannot exclude that the bacteria were unevenly distributed in the chocolate(products) and that those parts carrying many viable cells were not tested quantitatively. In chocolate, the low moisture (water activity a_w_: 0.4–0.5) and high sugar content does not favor bacterial growth, but significantly increases thermal resistance [11,18,19]. In addition, it has been speculated that the food matrix protects *Salmonella *against the acidic conditions of the stomach [11], which could imply that only few salmonellae are necessary to cause illness.

**Table 3:** Overview of published chocolate outbreaks due to *Salmonella *contamination

**Year**	**Country**	**Serovar**	Vehicle*	**Source of contamination**	**cfu/g**	**No. of affected persons**	**Peak of outbreak**	**Age of** ** cases**
1970	Sweden	*S*. Durham	Chocolate products (n>1),	Cocoa powder	/	110	Dec-May	53% ≤15 years
1973 – 1974	USA, Canada	*S*. Eastbourne	Chocolate balls from Canada	Cocoa beans	2.5	200	Dec-Feb.	3 years (median)
1982	England, Wales	*S*. Napoli	Chocolate bars from Italy	Unknown	2–23	272	May-Aug	58% ≤ 15 years
1985 – 1986	Canada	*S*. Nima	Chocolate coins from Belgium	Unknown	/	/	Dec-Jan	?
1987	Norway, Finland	*S*. Typhimurium	Chocolate products, (n = 3) from Norway	Avian contamination speculated	≤1	349	Mar-May	6 years (median)
2001– 2002	Germany, other European countries	*S*. Oranienburg	Two chocolate brands from Germany	Unknown	1.1–2.8	439	Oct-Dec	15 years (median)

* In each outbreak, the identified vehicles were traced to a single manufacturer

Company A produced several dozen tons of chocolate per day. All positive samples were produced in the same week. However, *S*. Oranienburg reports above an expected baseline of 1–2 reports per week in Germany were received for five months. The protracted nature of chocolate-associated outbreaks probably reflects both the long shelf-life of chocolate [20] and the long survival of *Salmonella *in these products [15,21]. *S*. Oranienburg was isolated from chocolates five months after manufacture. In an *S*. Napoli outbreak in England and Wales, this interval was 12 months. The number of affected persons reported in chocolate-associated *Salmonella *outbreaks has grown steadily over the years (table 3). Among other factors, this may parallel advances in food-processing technologies and improvements of national surveillance systems. Taken together, the chocolate industry faces a difficult situation because:

 raw ingredients (e.g., cacao beans, milk powder) can carry *Salmonella* spp.,

 the low water activity and high fat content in chocolate increases thermal resistance so that temperatures reached during chocolate production (even after considerable overheating [19]) do not necessarily destroy *Salmonella*,

 a small number of *Salmonella *may be sufficient to cause disease,

 even with low-level contamination, chocolate can affect large number of persons (often children) scattered over a wide area, and thus, has the potential to cause serious public health consequences.

It is noteworthy that the case-control study did not identify further products as risk factors. This applied also to the second contaminated brand of company A, which was included in the recall. The small proportion of study cases (25%) mentioning having eaten chain-X-chocolate lends support to the hypothesis of more contaminated brands (even from other manufacturers), which could be one explanation for the continuing case-occurrence. However, inaccuracies due to lack of brand awareness may have played a particular role in this outbreak, and the time-delay between disease onset and interview (median: 37 days) may have contributed to an inaccurate recall of cases and their guardians. Furthermore, cases with a disease onset in 2002 may have occurred as a result of a diminished impact of the public warning due to the Christmas season. For example, chocolate gifts received or given for Christmas may not have been thoroughly enough checked for best-before dates stated in the public warning.

Identification of vehicles in foodborne outbreaks can become difficult if the exposure is common. Consumption of a wide variety of German chocolates was reported by all case-patients (and 88% of explored patients), but also from 86% of the control subjects in the week prior to onset of illness. When groups are (nearly) universally exposed or a more specific hypothesis cannot be tested, often the best one can do is to establish a "dose-response-relationship" [22], i.e., unravel differences in the frequency of consumption of the incriminated food between cases and controls. Consequently, the observation that a higher proportion of cases reported eating chocolate on a daily basis added to the evidence that chocolate was the vehicle in this outbreak. Furthermore, the Danish data provided powerful supplementary evidence because consumption of German chocolate was particularly common in Germany but unusual in Denmark. Therefore, in multinational outbreaks, international collaboration provides an important means for disclosing the common source of infections, particularly when the contaminated food is very popular in one (likely the source) country (e.g., [23,24]). Multinational collaboration facilitated by Enter-net helped in preventing contaminated chocolate from entering the market in Canada, Finland and Sweden, thereby averting human illness. Furthermore, by rapid electronic exchange and comparison of PFGE profiles, the Canadian cluster of human cases could be classified as unrelated to this outbreak.

No source or point of contamination was identified. Hygienic deficiencies had not been observed at the production facility of company A, which used a modern production method. This included an extra heating of the milled cocoa beans by a special heat-steam treatment with 125–130°C as an additional safeguard. Samples from in-house chocolates and from ingredients tested negative. However, no environmental samples and very few samples of raw ingredients (n = 10) were obtained. In a *S*. Eastbourne outbreak in Canada/USA in 1973/74, 286 environmental samples and 98 chocolate samples from the production-line were examined. No in-line chocolate sample tested positive and overall only 6 (1.6%) samples were positive (bean processing rooms [n = 4], and samples from a molding plant [n = 2]] [12]. Therefore, source investigations in chocolate-outbreaks should include extensive sampling in the production environment to increase the likelihood of determining possible points of contamination. In this outbreak, it remains unclear whether the salmonellae survived the heating or (re)contaminated the chocolate afterwards. Consequently, long-term preventive measures to render chocolate-production safer could not be implemented.

An Enter-net urgent inquiry was sent after the first results of molecular subtyping suggested a link between human cases and chocolate from company A. Until then, investigators in Germany and Denmark had worked independently unaware that the outbreak extended outside of their respective countries. An earlier inquiry, ideally as early as an outbreak was suspected by the investigating countries (or as an increase was noted in the Enter-net database), may have speeded up hypothesis generating, and thus, may have helped in earlier identification of the vehicle, thereby preventing illnesses.

Finally, a public warning or recall of company A products did not occur before a brand A leftover tested positive although the confluence of information – the results of the case-control study, the Danish investigations, and the subtyping comparison between human isolates and the in-house sample – had already pointed to company A products as the source of the outbreak. Yet, no specific product or lot had been identified at the time. For this reason, a recall or a public warning were considered excessive responses by the German food safety authority. However, relying on microbiological confirmation in leftovers, if available for testing, is disputable (directionality of contamination unclear) and is dangerous in unopened food packages because critical time can elapse before a positive culture in food is obtained [25]. Therefore, it has been argued that public health action should be based on well-performed epidemiological investigations encompassing clear statistical associations with a specific exposure [25-27]. Such data are easiest to obtain when only one (ideally distinct) vehicle is involved that is infrequently consumed. Nonetheless, when food-production leads to more than one contaminated foodstuff, or when popular foods are vehicles of infection, hypothesis generating or testing can become intricate. Unfortunately, these instances appear conducive to affect large areas/populations. Therefore, we believe that clear associations even with surrogates of exposure suffice to justify public health actions (e.g., extensive source investigations) provided they plausibly fit other lines of evidence.

## Conclusions

To our knowledge, this is the largest reported chocolate-associated outbreak, the seriousness being emphasized by the hospitalizations (29%) and self-reported bloody diarrhea (21%) of the study cases. Despite the use of improved production technologies, the chocolate industry continues to carry a small risk of manufacturing *Salmonella*-containing products. For the future, awareness among German food safety authorities must be heightened for the need to base public health action not exclusively on laboratory confirmation in food, and to conduct timely and comprehensive source investigations to enhance food safety in the long-run. The international scale of this outbreak shows how easy it is to distribute a contaminated product across many countries. This underlines the necessity of mechanisms for international surveillance and information dissemination such as Enter-net to ensure that international outbreaks can be dealt with rapidly and in an appropriate manner. Similar networks should be set up or, if existing, should be connected (possibly overseen by WHO), to allow rapid communications to other parts of the world when it is clear that a contaminated product is distributed internationally.

## Competing interests

The author(s) declare that they have no competing interests.

## Authors' contributions

DW was the principal investigator of the German part of this outbreak; he carried out the statistical analysis of the case-control study, and drafted the manuscript. JD and FF were responsible for the outbreak investigation including the case-control study for the state ("Bundesland") of Lower Saxony. UvT was responsible for the outbreak investigation including the case-control study for the state ("Bundesland") of Northrhine-Westfalia. GF was responsible for the outbreak investigation including the case-control study for the state ("Bundesland") of Hamburg. SE conducted the Danish part of this outbreak investigation. AMH was responsible for the outbreak investigation including the case-control study for the state ("Bundesland") of Hesse. PR detected the outbreak and conducted microbiological investigations. RP and HT conducted the PFGE-analysis. ISTF coordinated the Enter-net inquiries and investigations. SB conducted interviews in the case-control study, designed the database and helped in the analysis. EB conducted quantitative analysis of *Salmonella *in chocolate. EW coordinated food safety investigations in this outbreak. AE conducted the Canadian part of this outbreak investigation. AS conducted the Finnish part of this outbreak investigation. YA conducted the Swedish part of this outbreak investigation. MHK was instrumental in the design of the case-control study. AA coordinated the German part of this outbreak investigation, broadened the scope of this outbreak by prompting an urgent Enter-net inquiry, and helped in designing the case-control study and drafting the manuscript.

All authors participated in revising the manuscript.

## Pre-publication history

The pre-publication history for this paper can be accessed here:


